# Liver-Specific Deletion of Phosphatase and Tensin Homolog Deleted on Chromosome 10 Significantly Ameliorates Chronic EtOH-Induced Increases in Hepatocellular Damage

**DOI:** 10.1371/journal.pone.0154152

**Published:** 2016-04-28

**Authors:** Colin T. Shearn, David J. Orlicky, Rebecca L. McCullough, Hua Jiang, Kenneth N. Maclean, Kelly E. Mercer, Bangyan L. Stiles, Laura M. Saba, Martin J. Ronis, Dennis R. Petersen

**Affiliations:** 1 Skaggs School of Pharmaceutical Sciences and Pharmacy, University of Colorado Anschutz Medical Campus, Aurora, CO, United States of America; 2 Department of Pathology, School of Medicine University of Colorado Anschutz Medical Campus, Aurora, CO, United States of America; 3 Department of Pediatrics, School of Medicine University of Colorado Anschutz Medical Campus, Aurora, CO, United States of America; 4 Arkansas Children's Nutrition Center, University of Arkansas for Medical Sciences, Little Rock, AK, United States of America; 5 Department of Pharmacology and Pharmaceutical Sciences, School of Pharmacy, University of Southern California, Los Angeles, CA, United States of America; INRA, FRANCE

## Abstract

Alcoholic liver disease is a significant contributor to global liver failure. In murine models, chronic ethanol consumption dysregulates PTEN/Akt signaling. Hepatospecific deletion of phosphatase and tensin homolog deleted on chromosome 10 (PTEN^LKO^) mice possess constitutive activation of Akt(s) and increased *de novo* lipogenesis resulting in increased hepatocellular steatosis. This makes PTEN^LKO^ a viable model to examine the effects of ethanol in an environment of preexisting steatosis. The aim of this study was to determine the impact of chronic ethanol consumption and the absence of PTEN (PTEN^LKO^) compared to Alb-Cre control mice (PTEN^f/f^) on hepatocellular damage as evidenced by changes in lipid accumulation, protein carbonylation and alanine amino transferase (ALT). In the control PTEN^f/f^ animals, ethanol significantly increased ALT, liver triglycerides and steatosis. In contrast, chronic ethanol consumption in PTEN^LKO^ mice decreased hepatocellular damage when compared to PTEN^LKO^ pair-fed controls. Consumption of ethanol elevated protein carbonylation in PTEN^f/f^ animals but had no effect in PTEN^LKO^ animals. In PTEN^LKO^ mice, overall hepatic mRNA expression of genes that contribute to GSH homeostasis as well as reduced glutathione (GSH) and oxidized glutathione (GSSG) concentrations were significantly elevated compared to respective PTEN^f/f^ counterparts. These data indicate that during conditions of constitutive Akt activation and steatosis, increased GSH homeostasis assists in mitigation of ethanol-dependent induction of oxidative stress and hepatocellular damage. Furthermore, data herein suggest a divergence in EtOH-induced hepatocellular damage and increases in steatosis due to polyunsaturated fatty acids downstream of PTEN.

## Introduction

Alcoholic liver disease (ALD) and non-alcoholic fatty liver disease (NAFLD) are two of the leading causes of liver disease in the United States today. Both ALD and NAFLD are characterized by progressive hepatocellular damage manifested in increased steatosis, steatohepatitis, fibrosis and ultimately progression to cirrhosis [1]. In the western world, the prevalence of diet-induced nonalcoholic steatohepatitis (NASH) has dramatically increased in the last decade. According to the latest data from the Centers of Disease Control, current estimates indicate 30–35% of all Americans are obese and 69% are overweight (http://www.cdc.gov/nchs/fastats/obesity-overweight.htm). Moderate alcohol consumption by human subjects with increased body mass index is strongly correlated with increased hepatic damage as determined by plasma alanine amino transferase (ALT) and Gamma-Glutamyl Transferase levels [2]. Given these statistics, obesity and its concomitant steatosis are predictable cofactors for ALD.

In the liver, insulin is a major regulator of lipid metabolism and alteration of insulin signaling can induce hepatocellular lipid accumulation evident in both ALD and NAFLD [3–7]. Downstream of the insulin receptor, the PTEN/Akt pathway regulates insulin signaling [8]. Recruitment and full activation of Akt requires interaction of N-terminal pleckstrin homology domains with phosphatidylinositol (3,4,5) trisphosphate (PIP_3_) on intracellular membranes [9, 10]. Through its ability to catalyze the hydrolysis of the 3’ position phosphate on PIP_3_, PTEN negatively regulates Akt activation and insulin signaling [11]. This is highlighted by bypassing the Akt arm of insulin receptor signaling using mice containing a hepatospecific deletion of PTEN (PTEN^LKO^). PTEN^LKO^ mice possess constitutive Akt activation, hepatic insulin hypersensitivity and increased steatohepatitis [12–14]. In our previous study using the PTEN^LKO^ model, we determined that increased consumption of a diet high in polyunsaturated fatty acids potentiates hepatocellular damage and decreases hepatocellular redox capacity when compared to chow fed controls [15].

During chronic alcohol consumption, there is pronounced lipid accumulation and enhanced oxidative stress [16]. In murine models of ALD, increased *de novo* lipogenesis (DNL) has been proposed to be a contributing factor in lipid accumulation [17, 18]. Chronic consumption of EtOH combined with dietary polyunsaturated fatty acids decreases PTEN expression/activity increasing activation of Akt2 [19]. In other models, chronic EtOH consumption has also been demonstrated to enhance the Akt activated transcription factor SREBP1, increasing fatty acid synthesis [17]. Furthermore, using a short term model of EtOH toxicity, pre-administration of insulin reduced oxidative stress and hepatocellular damage but did not diminish steatosis further demonstrating the contribution of insulin signaling in EtOH toxicity [20]. Most recently, an environment of increased insulin hypersensitivity created by PTEN^LKO^ was demonstrated to provide protection against murine endotoxemia [21]. Thus, it would be reasonable to predict that if insulin signaling was completely bypassed by using mice possessing a constitutively activated form of Akt, then EtOH administration would not increase hepatocellular damage but still increase steatosis. In the present study, mice possessing constitutively activated Akt (PTEN^LKO^) were used in a well characterized 6-week model of chronic EtOH consumption to further elucidate the contribution of PTEN/Akt signaling in EtOH-induced steatosis and hepatotoxicity. In PTEN^LKO^ mice, chronic EtOH consumption did not increase hepatocellular damage and corresponded with elevated glutathione metabolism. We hypothesize that the aforementioned elevation in glutathione metabolism assists in mitigation of hepatocellular damage induced by EtOH.

## Materials and Methods

### Animal Model

To generate liver-specific PTEN deletion mice (PTEN^LKO^), mice carrying PTEN conditional knockout alleles (PTEN^loxP/loxP^; Alb-Cre^−^ (PTEN^f/f^)) were bred with an Albumin (Alb)-Cre-recombinase transgenic mouse as previously described [12, 15]. At 5 weeks of age, tail snips were taken and mice were genotyped using PTEN^f/f^ and Cre specific primers (Transnetyx, Memphis TN). At 12 weeks of age, mice were isocalorically pair fed (PF) in groups of 6 either a modified Lieber-DeCarli diet (45% polyunsaturated fat derived calories mostly from corn oil) (Bio-Serv, Frenchtown, NJ) or EtOH-fed (EtOH-derived caloric content with an initial EtOH concentration of 10.8%, which was subsequently increased weekly to 16.2, 21.5, 26.9, 29.2, 31.8% ethanol derived calories for a total of 6-weeks. [19, 22]. Upon completion of the study, animals were anesthetized via intraperitoneal injection with sodium pentobarbital and euthanized by exsanguination. Blood was collected from the inferior vena cava and plasma was separated through centrifugation at 4°C and assayed for ALT activity (Sekisui Diagnostics, P.E.I., Canada). Excised livers were weighed, homogenized and subjected to differential centrifugation and subcellular fractionation (cytosolic, mitochondrial, microsomal and nuclear fractions) as previously described [23]. All procedures involving animals were approved by the Institutional Animal Care and Use Committee of the University of Colorado and were performed in accordance with published National Institutes of Health guidelines.

### Western Blotting

Proteins from either whole liver extracts or subcellular fractions were subjected to standard SDS-PAGE and transferred to PVDF (GE Healthcare, Picataway, NJ). Membranes were processed using the following antibody dilutions: ALDH2, 1:2000, Cat #15310 (Protein Tech Rosemont, IL.), AHD1, 1:1000 Cat#GTX62515 (Genetex, Irvine, CA.), beta actin 1:5000, Cat#A5441 (SIGMA, Saint Louis, MO) as previously described [15, 19, 22, 24]. Chemiluminescence was visualized using either film or a Storm 860 scanner from Molecular Dynamics (Sunnyvale, CA).

### Biochemical Analysis

Liver triglycerides were measured using a 2:1 chloroform:MEOH extract of liver homogenate using a kit from Diagnostic Research Inc. Protein concentrations were determined using a modified Lowry Protein Assay from Bio-Rad (Hercules, CA). Blood ethanol concentrations were determined from freshly isolated serum as previously described. [25, 26]

Microarray analysis: For microarrays, total RNA was extracted from fresh frozen pooled tissue isolated from triplicates of PF/EtOH-fed PTEN^f/f^/PTEN^LKO^ mice. Following transcription, cDNA was processed and analyzed as previously described [22, 27].

### Immunohistochemistry

Sections of freshly excised liver tissue were placed in 10% neutral buffered formalin (SIGMA) for 16 hours, followed by incubation in 70% EtOH overnight. Samples were then processed, embedded in paraffin, cut and sections were mounted on slides by the UC Denver Histology Core. Immunohistochemical characterization was performed using rabbit polyclonal antibodies directed against acrolein (Dilution 1:250, Cat#PA2049, Cell Sciences, Canton, MA), 4-HNE (Dilution 1:500), MDA (Dilution 1:100) and CYP2E1 (Dilution 1:750, Cat#1252, Millipore, Billerica, MA), Protein-SSG (Dilution 1:100, Cat#101-A-100, Virogen, Watertown, MA) as described [19, 24, 28]. Histologic images were captured on an Olympus BX51 microscope equipped with a four megapixel Macrofire digital camera (Optronics; Goleta, CA) using the PictureFrame Application 2.3 (Optronics).

### Statistical Analysis

Relative densitometry of Western blots was quantified using ImageJ (http://rsb.info.nih.gov/ij/). All data and statistical analysis was performed by two-way Analysis of Variance or a students t-test using Prism 5 for Windows (GraphPad Software, San Diego, CA). All data are expressed as mean +/- S.E.M. and *p* values <0.05 were considered significant.

## Results

### Chronic EtOH consumption increases Akt phosphorylation in WT mice but has no further effect in PTEN^LKO^ mice

To verify deletion of PTEN in our model, hepatic cytosolic extracts were prepared from PF and EtOH-fed PTEN^f/f^ and PTEN^LKO^ mice. As shown in S1A and S1B Fig, EtOH consumption decreased overall PTEN expression and increased Akt phosphorylation in PTEN^f/f^ mice. In PF PTEN^LKO^ mice, PTEN expression was decreased by greater than 95% and cytoplasmic pSer^473^Akt levels significantly increased by 7-fold above normal. Addition of EtOH did not significantly affect total cytosolic levels of or phosphorylation of the cytosolic Akt in the PTEN^LKO^ mice. Combined these data support constitutive activation of Akt’s in PTEN^LKO^ mice irrespective of EtOH addition.

### Deletion of PTEN confers significant protective effects against EtOH induced liver injury. Effects of PTEN^LKO^ and EtOH on hepatocellular function

We have previously demonstrated the consumption of a diet rich in polyunsaturated fatty acids exerts an additive effect with respect to hepatocellular damage in PTEN^LKO^ mice. The data presented in Table 1 presents the effects of either a PF diet or PTEN^LKO^ on EtOH induced hepatotoxicity. In the PTEN^LKO^ PF animals, serum ALT increased 10.7-fold when compared to PTEN^f/f^ animals. In the PTEN^f/f^ animals, chronic EtOH resulted in a 2.18-fold increase in serum ALT, a result comparable to values obtained using the C57BL6/J strain [22]. Interestingly, EtOH ingestion in the PTEN^LKO^ background resulted in a significant (p<0.05) 42% decrease in ALT relative to the PTEN^LKO^ pair fed group. Comparing liver to body weight ratios, EtOH consumption significantly increased liver/body weight in the PTEN^f/f^ group. Compared to PF PTEN^f/f^ mice, liver to body weight was 3.2-fold higher in the PTEN^LKO^ PF animals. The addition of EtOH did not have a further effect on liver to body weight ratio in PTEN^LKO^ animals. As expected, under conditions of sustained lipid synthesis, hepatic triglycerides were elevated by 6-fold in the PF PTEN^LKO^ animals compared PF PTEN^f/f^ groups [12, 29]. In the EtOH-fed groups, a significant increase in hepatic triglycerides in occurred in PTEN^f/f^ animals but triglycerides significantly decreased in EtOH-fed PTEN^LKO^ animals when compared to PF PTEN^LKO^ animals (p = 0.05). Overall, 2-way ANOVA indicated a significant interaction between PTEN^LKO^ and EtOH with respect to ALT, liver triglycerides and liver to body weight. In summary, when compared to the PTEN^LKO^ PF group, chronic EtOH consumption results in decreased hepatocellular damage as evidenced by decreased ALT and hepatic triglycerides in PTEN^LKO^ mice.

**Table 1:** Biochemical analysis of liver homogenates of PF and EtOH PTEN^f/f^ and PTEN^LKO^ mice.

Parameter^╪^	Pair-fed	EtOH	Pair-fed	EtOH	Genotype	EtOH	Interaction
*ALT (U/L)*	16.70±3.235^a^	36.44±5.604^b^	178.47±24.04^a,c^	103.98±11.62^b,c^	**<0.0001**	**0.0490**	**0.0022**
*Liver weight*	0.94±0.05^a^	1.08±0.05^b^	2.63±0.21^a,c^	2.22±0.07^b,c^	**<0.0001**	**0.0261**	0.2675
*Body weight*	29.87±0.20^a^	27.15±0.19^b^	25.61±0.77^a,c^	22.37±0.45^b,c^	**<0.0001**	**<0.0001**	0.5786
*Change in body weight*	6.39±0.73^a^	2.21±0.40^b^	3.83±0.39^a^	0.37±0.59^b^	**0.0004**	**<0.0001**	0.5125
*Liver/Body Weight*	3.20±0.11^a^	4.03±0.18^b^	11.16±0.31a,^c^	10.23±0.23^b,c^	**<0.0001**	0.8182	**0.0005**
*Liver Triglycerides (mmol/mg tissue)*	0.003±0.001^a^	0.008±0.001^b^	0.020±0.001^a,c^	0.017±0.001^b,c^	**<0.0001**	0.4005	**0.0062**
*Food Intake (mls/day)*	16.83±0.44	17.87±0.36	16.92±0.44	17.30±0.27	0.5462	0.0756	0.4031

Serum ALT, liver weight, body weight, change in body weight, liver to body weight, liver triglycerides and food intake were determined as described in methods. Data are means± SEM as analyzed by two-way ANOVA with a Bonferroni *post hoc* analysis (PTEN^f/f^ group compared to PTEN^LKO^ group). Means without a common superscript letter are significantly different (N = 7 pairs of PTEN^f/f^ PF/EtOH and 7 pairs of PTEN^LKO^ PF/EtOH mice/group (p<0.05)). Values in bold indicate significance by two-way ANOVA.

^╪^Letter superscripts (a,b,c) denote significant difference of P<0.05 by unpaired t-test, values in bold significant difference by Two-Way ANOVA.

### Chronic EtOH consumption decreases periportal steatosis in PTEN^LKO^ mice

**Fig 1:**
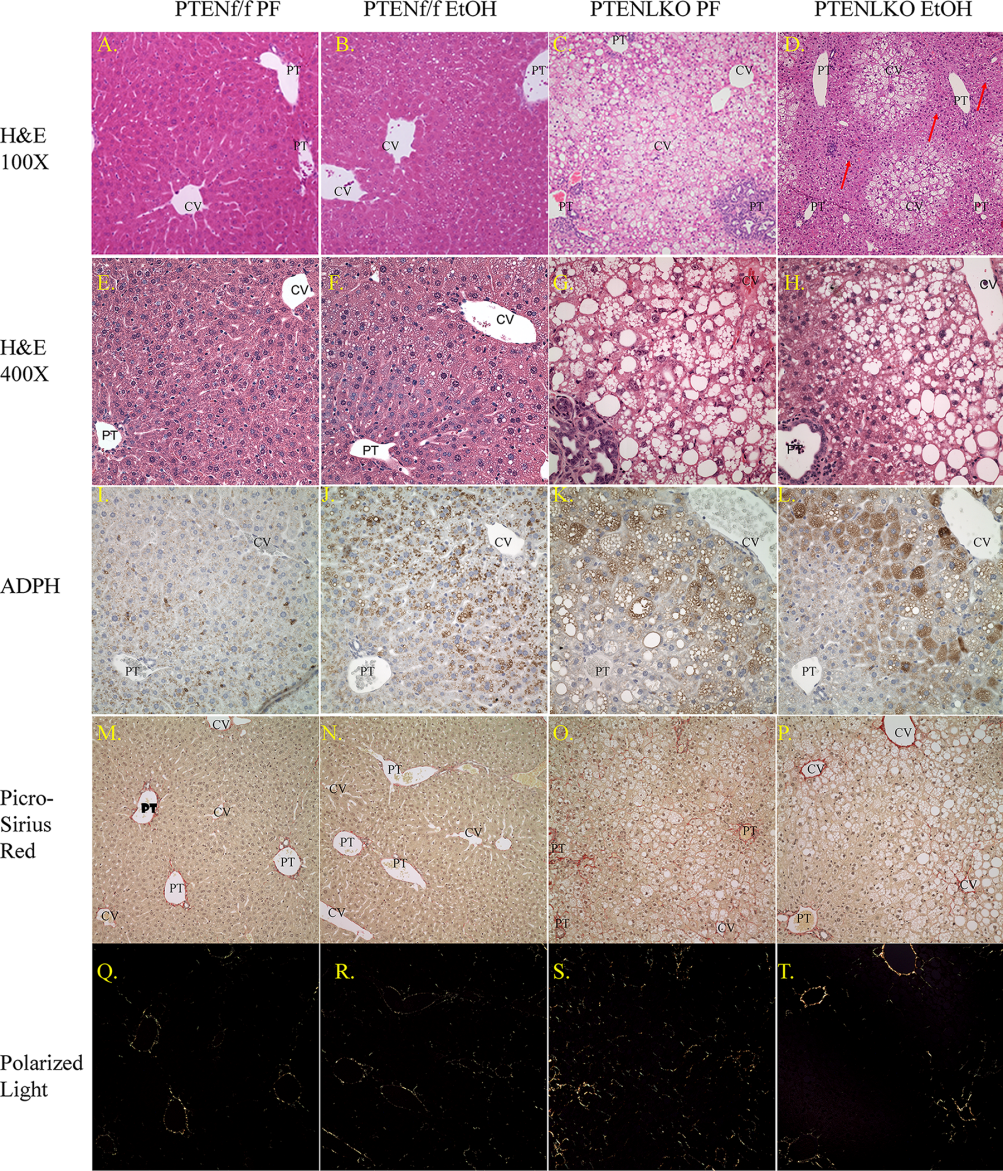
Effects of chronic EtOH on hepatic lipid accumulation and fibrosis in PTEN^f/f^ and PTEN^LKO^ mice. Panels: (A-D), hematoxylin and eosin staining (100X) of liver sections isolated from PF/EtOH PTEN^f/f^ and PTEN^LKO^ mice (N = 7 pairs of PTEN^f/f^ PF/EtOH and 7 pairs of PTEN^LKO^ PF/EtOH mice/group), (E-H), (400X), (I-L), adipophilin staining (N = 3 pairs of PTEN^f/f^ PF/EtOH and 3 pairs of PTEN^LKO^ PF/EtOH mice/group), (M-P), picrosirius red staining of liver sections (N = 4 pairs of PTEN^f/f^ PF/EtOH and 3 pairs of PTEN^LKO^ PF/EtOH mice/group), (Q-T), polarized light exposure of picrosirius red staining. (CV, central vein, PT, portal triad). Original Magnification 400X, H&E staining, 100X, picrosirius red collagen staining.

Given the finding that the addition of EtOH resulted in a decrease in ALT, the effects of EtOH in the PTEN^LKO^ background was further explored with respect to hepatocellular pathology using histology. Using hematoxylin and eosin staining, at low magnification (100X), mild steatosis occurred in EtOH-fed PTEN^f/f^ mice that was barely visible (Fig 1 panels A-D). Steatosis was much more significant in liver sections from PTEN^LKO^ PF mice but steatosis decreased with EtOH (Arrows Panel D). To better assess specific changes in hepatic pathology a higher magnification was employed. At higher magnification (400X) (Fig 1 panels E-H), formation of mild steatosis is much more visible following consumption of EtOH in PTEN^f/f^ mice. In the PTEN^LKO^ mice, consumption of the high fat diet in the PF group induced severe panlobular steatosis. In agreement with hepatic triglyceride accumulation, EtOH consumption by the PTEN^LKO^ mice decreased steatosis primarily in the periportal region which parallels the observed decrease in hepatic triglycerides presented in Table 1. As an additional method to support the change in periportal lipid accumulation, tissues sections were stained with adipophilin. As shown in Fig 1 Panels I-L, compared to the PTEN^LKO^ PF group, adipophilin staining decreased in the periportal region of PTEN^LKO^ EtOH mice. These data support decreased periportal lipid accumulation in EtOH-fed PTEN^LKO^ mice.

To further explore differences in overall pathology in PTEN^LKO^ PF/EtOH fed mice, a modified Kleiner Scoring was performed on hematoxylin and eosin stained hepatic tissue sections [30]. As shown in Table 2, with the exception of a mild increase in steatosis, PTEN^f/f^ PF/EtOH groups did not exhibit significant differences in overall hepatic pathology. Not surprisingly, both PF and EtOH-fed PTEN^LKO^ groups exhibited increased biliary hyperplasia [12, 13]. Although there was an increasing trend, at least by pathology, steatosis was not significantly increased by EtOH in the PTEN^f/f^ control groups. Steatosis in the PTEN^LKO^ PF group was extensive and panlobular. Addition of EtOH resulted in a significant decrease in steatosis primarily in zone 1. Both PTEN^LKO^ groups exhibited macro and microsteatosis. Overall, the combined modified Kleiner score was significantly decreased by 6-weeks consumption of EtOH in PTEN^LKO^ mice. An examination by Two-Way analysis of variance revealed a significant interaction between the PTEN^LKO^ genotype and EtOH with respect to overall hepatic steatosis and overall modified Kleiner score.

**Table 2:** Modified Kleiner scoring of tissue sections isolated from PF and EtOH PTEN^f/f^ and PTEN^LKO^ mice.

		PTEN^f/f^			PTEN^LKO^			Two-Way ANOVA	
Parameter^╪^	PF		EtOH	PF		EtOH	Genotype	EtOH	Interaction
*Steatosis (0–3)*	0.04±0.12^a^		0.43±0.13^b^	2.93±0.13^a,c^		2.07±0.16^b,c^	**<0.0001**	0.0965	**0.0206**
*Ballooning/Degeneration (0–2)*	0.0±0.0		0.0±0.0	1.14±0.23^c^		0.43±0.28^c^	**0.0002**	0.0618	0.0618
*Lobular Inflammation (0–2)*	0.0±0.0		0.0±0.0	1.00±0.0^c^		1.00±0.0^c^	**<0.0001**	1	1
*Microsteatosis (0–3)*	0.0±0.0		0.0±0.0	1.00±0.0^c^		1.00±0.0^c^	**0.0001**	1	1
*Score Modified Kleiner (0–10)*	0.04±0.12^a^		0.43±0.13^b^	6.07±0.25^a,c^		4.50±0.30^b,c^	**<0.0001**	**0.0099**	**0.0002**
*Biliary Hyperplasia*	0.0±0.0		0.0±0.0	2.00±0.29^c^		2.14±0.23^c^	**<0.0001**	0.7086	0.7068

Overall hepatic steatosis, hepatocyte ballooning, lobular inflammation, microsteatosis, biliary hyperplasia were quantified according to Brunt and Kleiner with minor modifications **[30]**. Data are means± SEM as analyzed by two-way ANOVA with a Bonferroni *post hoc* analysis (PTEN^f/f^ group compared to PTEN^LKO^ group). To determine specific effects of EtOH within each group, an unpaired student’s t-test was performed. Means without a common superscript letter are significantly different (N = 7 pairs of PTEN^f/f^ PF/EtOH and 7 pairs of PTEN^LKO^ PF/EtOH mice/group (p<0.05)). Values in bold indicate significance by two-way ANOVA.

^╪^Letter superscripts (a,b,c) denote significant difference of P<0.05 by unpaired t-test, values in bold significant difference by Two-Way ANOVA.

In a previous report, PTEN^LKO^ mice develop fibrosis by 40 weeks [12, 13]. Although the mice used in the present study were only 18 weeks old at study completion, we hypothesized that EtOH may also affect fibrosis. Therefore, we examined the effects of 6 weeks of EtOH consumption on fibrosis using Picrosirius red staining [31]. To eliminate showing “selected” fields of the liver, lower magnification are presented in Panels M-P (white light) and lower panels ((Panels Q-T) polarized light). Chronic EtOH consumption did not result in an increase in picrosirius red staining in the PTEN^f/f^ animals. In PF PTEN^LKO^, only mild fibrosis was present compared to PF PTEN^f/f^ mice. Following 6-weeks consumption of EtOH, no significant differences were evident in PTEN^LKO^ mice (quantification not shown), indicating that fibrosis as evidenced by collagen deposition was not affected by EtOH consumption in PTEN^LKO^ mice.

### Effects of PTEN^LKO^ and EtOH on ADH and ALDH2 expression

**Fig 2:**
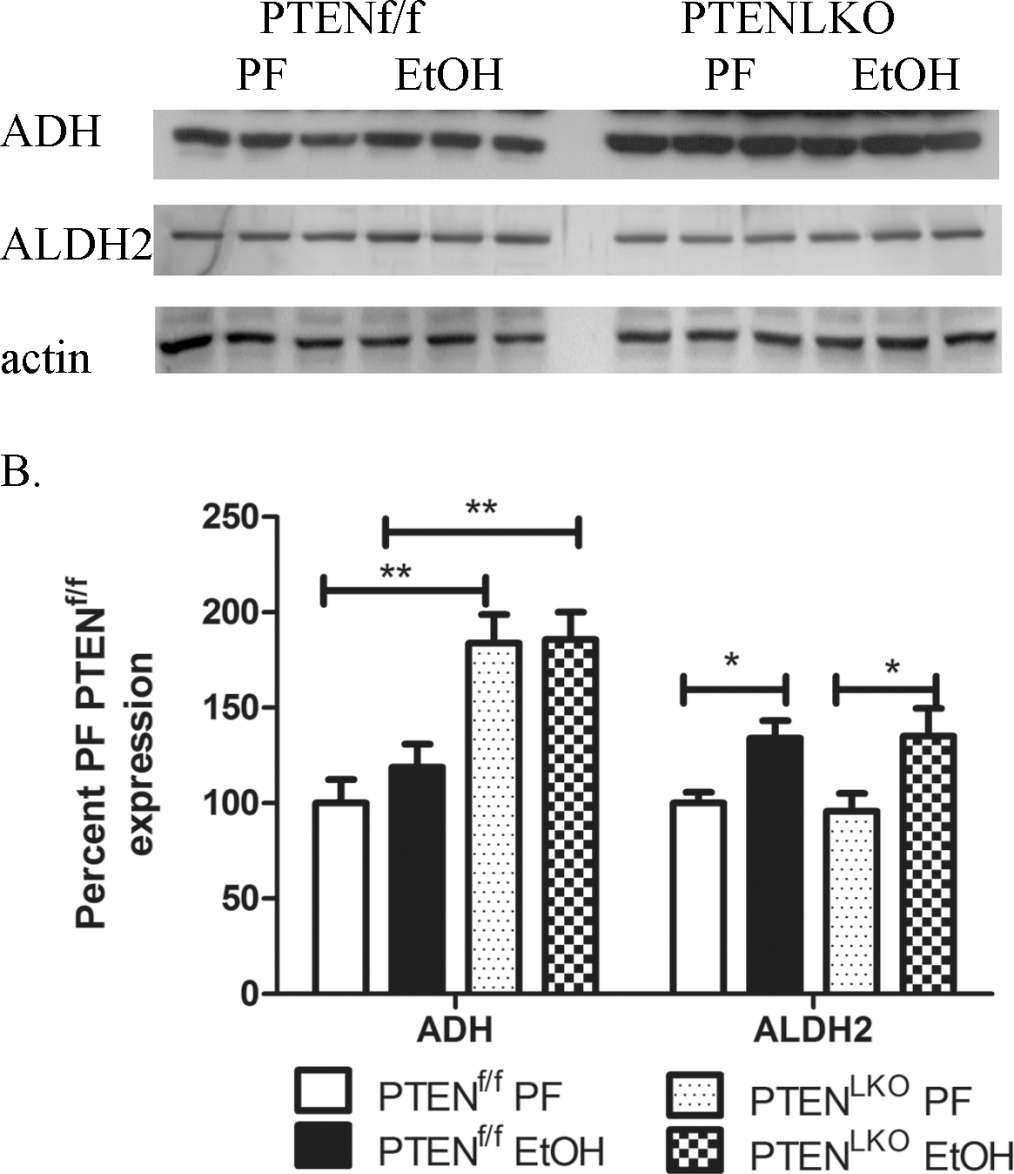
Effects of PTEN^LKO^ and EtOH on expression of EtOH metabolizing enzymes. Whole cell extracts fractions from PF control and EtOH PTEN^f/f^/PTEN^LKO^ groups were analyzed via SDS PAGE, Western blotted and probed for ADH and ALDH2. (A) Western blotting analysis of ALDH2 and ADH expression in PF and EtOH-fed PTEN^f/f^ and PTEN^LKO^ mice. (B) Quantification of the Western blots presented in Fig 2A (actin normalized). Data are means± SEM as analyzed by students t-test (PF/EtOH) and two-way ANOVA with a Bonferroni *post hoc* analysis (PTEN^f/f^ group compared to PTEN^LKO^ group) (N = 6 mice/group (*p<0.05, ***p<0.001)).

When ingested, EtOH is first metabolized by alcohol dehydrogenase 1 (ADH1) forming acetaldehyde which is then further metabolized by aldehyde dehydrogenase 2 (ALDH2) to produce acetate [32]. To determine the effects of PTEN^LKO^ on EtOH metabolism, expression of ADH1, and ALDH2 was examined. As shown in Fig 2A and 2B, EtOH ingestion significantly increased ALDH2 expression in PTEN^f/f^ and PTEN^LKO^ mice. Chronic EtOH consumption had no effect on ADH expression in the PTEN^f/f^ mice but in PTEN^LKO^, a significant increase was present in both PF and EtOH mice. This suggested that metabolism of EtOH might be increased in PTEN^LKO^ mice. Therefore, overall blood ethanol concentrations (BEC) were examined using serum isolated from each group. In EtOH-fed PTEN^f/f^ and PTEN^LKO^ mice, BEC was increased (PTEN^f/f^ 137.38±66.69, PTEN^LKO^ 120.25±26.48) but no significant differences were evident between the two genotypes indicating that metabolism of EtOH is not significantly affected by hepatospecific deletion of PTEN.

### Consumption of EtOH does not increase protein carbonylation in PTEN^LKO^ mice

**Fig 3:**
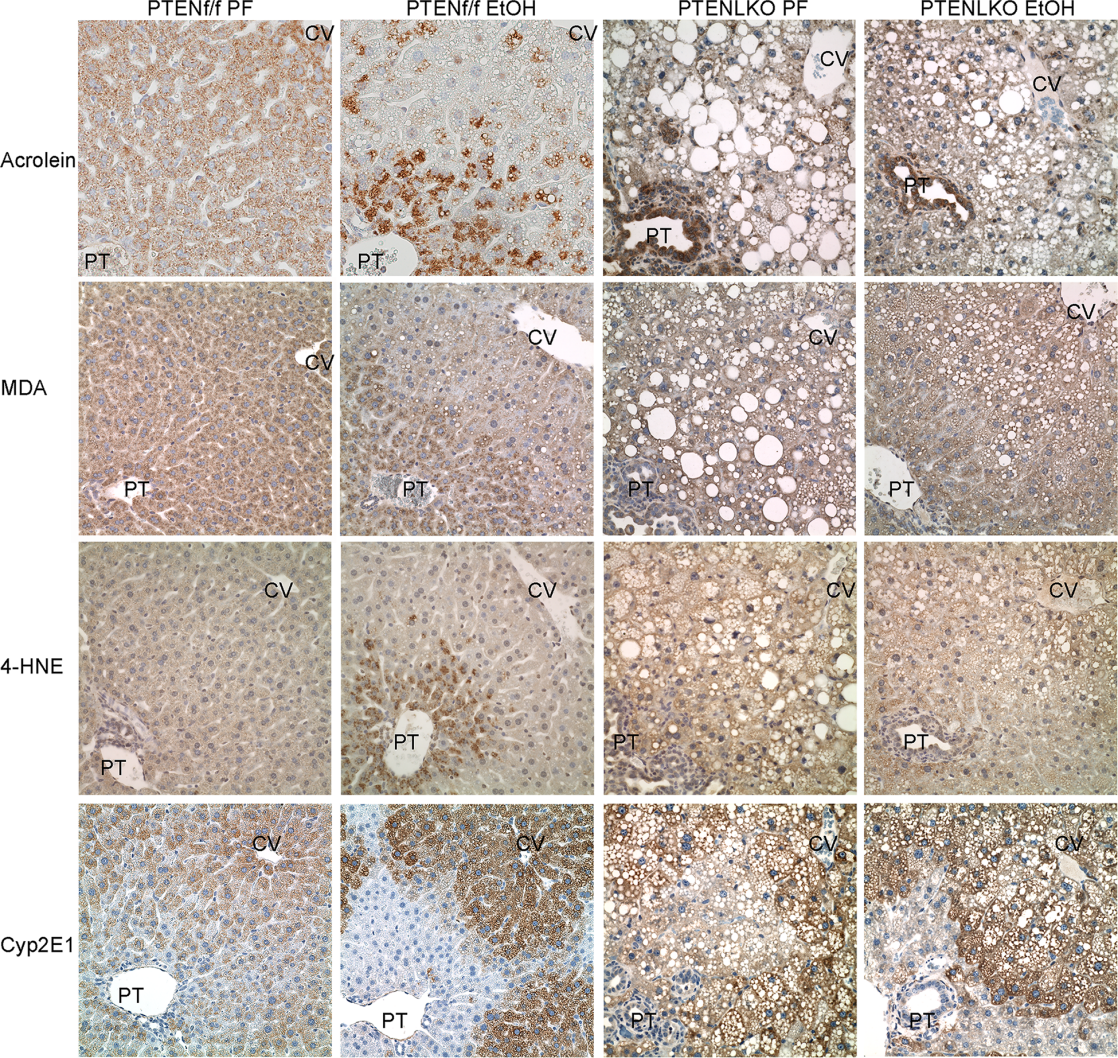
Effects of chronic EtOH on protein carbonylation in PTEN^LKO^ mice. (A) Hepatic tissue sections isolated from PF and EtOH-fed PTEN^f/f^/PTEN^LKO^ mice were analyzed for protein modification by acrolein, MDA and 4-HNE and Cyp2E1 expression using immunohistochemistry. Figures are representative of five PF/EtOH groups of PTEN^f/f^ and PTEN^LKO^ mice respectively.

Chronic EtOH consumption results in increased accumulation of lipid aldehyde modified hepatic proteins (protein carbonylation) [19, 33]. To examine the effects of constitutive Akt activation on protein carbonylation, liver sections prepared from PTEN^f/f^ and PTEN^LKO^ pairs were examined for expression of Cyp2E1, acrolein, 4-HNE and MDA via immunohistochemistry (Fig 3). Expression of Cyp2E1 was clearly elevated in the centrilobular region of both genotypes following EtOH consumption. Examining protein carbonylation, in PTEN^f/f^ animals, EtOH-induced a periportal increase in post-translational modification of proteins by oxidative stress induced lipid peroxidation products acrolein, MDA and 4-HNE. Surprisingly, in the PTEN^LKO^ model, this increase was not evident. As interesting, strong acrolein staining occurred in the cholangiocytes of PTEN^LKO^ mice although the ramifications of increased cholangiocyte acrolein staining are not clear. Collectively, these data indicate chronic consumption of EtOH results in Cyp2E1 induction that is not associated with increased protein carbonylation in PTEN^LKO^ mice.

### GSH homeostasis is increased in PTEN^LKO^ mice

**Fig 4:**
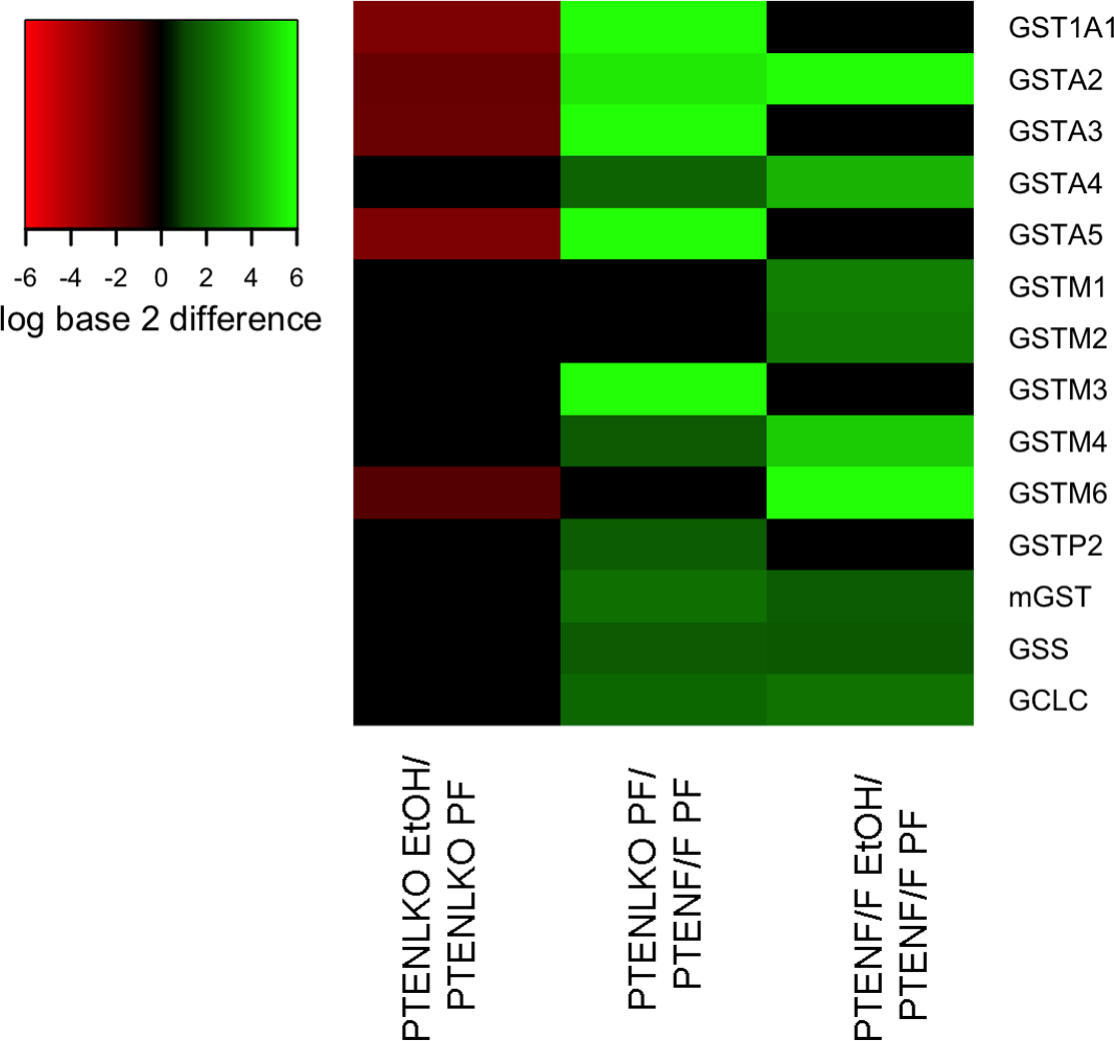
Effects of PTEN^LKO^ and EtOH on expression of GSH homeostatic genes. A limited microarray was performed using pooled tissue isolated from 3 pairs of PF/EtOH fed PTEN^LKO^ and PTEN^f/f^ respectively. The values represented in the heat map are log base 2 differences in gene expression compared to the respective control. The values are indicated by color with red representing lower expression in the following: PTEN^LKO^ EtOH (lane 1), PTEN^LKO^ PF (lane 2), PTEN^f/f^ EtOH (lane 3), compared to the control: PTEN^LKO^ PF (lane 1), PTEN^f/f^ PF (lane 2), PTEN^f/f^ PF (lane 3) group and with bright green representing higher expression in the treatment groups compared to the controls. Differences in expression that were between -1.5 and 1.5 are represented using the color black.

In mice, 6-weeks consumption of EtOH results in a 30% decrease in total hepatic GSH concentrations [34]. By its ability to conjugate GSH with reactive aldehydes, glutathione S-transferase A4 (GSTA4) represents a primary mechanism for regulation of hepatic protein carbonylation [35–38]. To determine if alterations in GSH metabolism are contributing factors in mitigation of oxidative stress occurring in PTEN^LKO^ mice, tissue was isolated and pooled from 4 PF/EtOH fed PTEN^f/f^/PTEN^LKO^ mice respectively. Microarray analysis comparing PF/EtOH fed PTEN^LKO^, PF PTEN^f/f^ /PF PTEN^LKO^ and PTEN^f/f^ PF/EtOH fed was then performed using cDNA transcribed from mRNA. From the heat map presented in Fig 4, chronic EtOH administration increased mRNA expression of GSTa2/4, GSTm1/2/4/6, glutathione synthetase (GSS) and glutathione cysteine ligase (GCLC). When compared to the PF PTEN^f/f^ group, PF PTEN^LKO^ mice exhibited increased mRNA expression of almost all GSTs as well as GSH synthesizing enzymes. Addition of EtOH decreased GSTa1/2/3/5 and GSTm6 in PTEN^LKO^ mice. Overall these data support increased GSH homeostasis in PTEN^LKO^ mice. KEGG pathways analysis was used to gain addition understanding of the ramifications of changes in the genes presented in Fig 4. From the data presented in S1 Table, hepatospecific deletion of PTEN significantly affected glutathione metabolic and xenobiotic detoxification pathways.

### Effects of PTEN^LKO^ and EtOH on overall glutathione oxidation and reduction (redox) capacity

**Fig 5:**
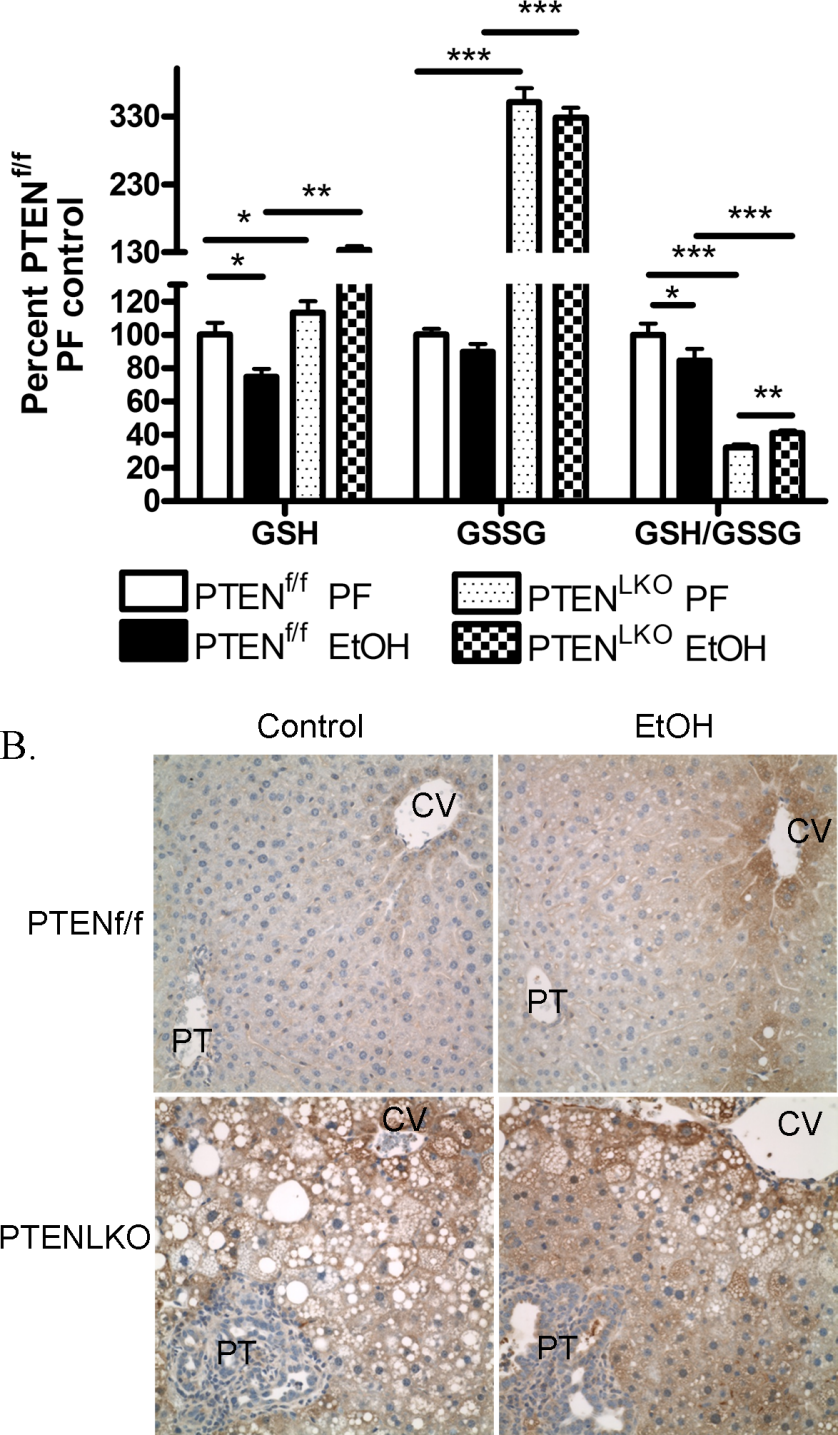
Oxidative stress measurements in hepatic tissue isolated from PF and EtOH-fed PTEN^f/f^ and PTEN^LKO^ mice. (A) GSH, GSSG and GSH/GSSG. Total hepatic concentrations of GSH and GSSG were determined from 6 pairs of PF/EtOH-fed PTEN^f/f^ and 7 pairs of PTEN^LKO^ respectively. Data are means± SEM as analyzed by students t-test (PF/EtOH) and two-way ANOVA with a Bonferroni *post hoc* analysis (PTEN^f/f^ group compared to PTEN^LKO^ group) (N = 6 mice/group (*p<0.05, ***p<0.001)) (B) Hepatic tissue sections isolated from PF and EtOH-fed PTEN^f/f^/PTEN^LKO^ mice were analyzed for Protein-SSG using immunohistochemistry. Figures are representative of 3 PF/EtOH groups of PTEN^f/f^ and PTEN^LKO^ mice respectively.

In hepatocytes, reactive aldehydes are removed via conjugation to GSH [39]. Oxidation of glutathione (GSSG) occurs under conditions of oxidative stress and a significant decrease in the ratio of GSH:GSSG is an accepted marker of increased oxidative stress [40, 41]. In wild type murine models, chronic addition of EtOH results in a decrease in total reduced GSH but no significant change in GSSG [34]. We hypothesized that in PTEN^LKO^ mice, an increase in GSH contributes to decreased carbonylation following chronic EtOH consumption. From Fig 5A, EtOH decreased GSH by 30% in the PTEN^f/f^ animals, a result similar to data previously obtained in WT C57BL6/J mice [16]. Comparing both genotypes, deletion of PTEN significantly increased GSH and GSSG. In the PTEN^LKO^ model, EtOH slightly increased hepatic GSH but did not affect GSSG. Examining redox status, the ratio of GSH:GSSG significantly decreased in PTEN^f/f^ mice following EtOH. This effect was reversed in PTEN^LKO^ mice. Compared to either group of PTEN^f/f^ mice, cellular redox ratios significantly decreased in the PTEN^LKO^ mice. Using 2-way ANOVA, genotype effects were evident for all three parameters and a significant interaction occurred with respect to GSH and redox ratio. Combined with data presented in Figs 1, 2 and 3, these data indicate that in PTEN^LKO^ mice there is increased GSH, enhanced mRNA expression of glutathione S-transferases correlating with mitigation of protein carbonylation, decreased ALT and decreased hepatic triglycerides.

### Protein glutathionylation is increased in PTEN^LKO^ mice

In the PTEN^LKO^ mice, there is a significant increase in GSH and GSSG. We hypothesized that increased cellular GSH would also result in an increased in protein glutathionylation. As shown in **Fig 5B**, in the PTEN^f/f^ group, protein-SSG is increased around the central vein following consumption of EtOH indicating an increase in oxidative stress. Examining PF/EtOH fed PTEN^LKO^ mice, staining of protein SSG was increased when compared to respective PTEN^f/f^ controls but no effect was evident with respect to EtOH.

## Discussion

Steatosis is an early consequence of ALD as well as NASH. Given that in the Western world, obesity and NASH are rapidly increasing it is critical to understand that combinatorial effects of alcohol and NASH. Recent research has clearly demonstrated that a major regulator of hepatocellular lipid accumulation is the PTEN/Akt pathway [12]. We previously demonstrated that in C57BL/6J WT mice, chronic consumption of EtOH increases PTEN phosphorylation, carbonylation and decreases PTEN expression [19]. This translated to an increase in Akt activation contributing to the formation of steatosis [19, 42]. To further elucidate the contribution of PTEN/Akt signaling in EtOH-induced hepatocellular toxicity, we utilized PTEN^LKO^ mice as a model of increased Akt activation and preexisting steatosis [11, 15, 19]. Our initial hypothesis was that hepatocellular damage would not increase following EtOH consumption and that concurrent steatosis in PTEN^LKO^ mice in conjunction with EtOH would exert an additive effect with respect to hepatic triglyceride accumulation. From the results obtained in this study, chronic consumption of EtOH reversed high fat diet induced increases in hepatocellular damage as evidenced by decreased ALT and also decreased hepatic triglycerides.

Compared to the pair-fed PTEN^f/f^ genotype, pair-fed PTEN^LKO^ mice displayed a dramatic 3-fold increase in overall liver weight, an increased liver:body weight ratios. Hepatic triglycerides and ALT both increased by 10-fold accompanied by a dramatic reduction of GSH/GSSG ratio. These data demonstrate that the high polyunsaturated fat PF diet produces significant liver injury by itself in the PTEN^LKO^ mice and is associated with increased oxidative stress and inflammation. In the PTEN^f/f^ animals, chronic EtOH consumption resulted in a mild but significant increase in hepatic triglycerides, steatosis and hepatocellular damage as shown by increased ALT as well as by increased protein carbonylation. In the PTEN^LKO^ model, surprisingly, chronic EtOH addition resulted in a significant decrease in ALT, periportal steatosis and hepatic triglycerides. Furthermore, compared to PF PTEN^LKO^, an increase in protein modification by reactive aldehydes did not occur. This is in agreement with previous data that demonstrates that PTEN^LKO^ mice are resistant to additional hydrogen peroxide-induced oxidative stress [43]. Furthermore, in chow-fed PTEN^LKO^ mice, basal oxidative stress is increased when compared to WT controls [44].

In the PTEN^LKO^ PF group, expression of GSH metabolizing enzymes (GST’s, GCLC, GSS) are increased when compared to PTEN^f/f^ controls. This corresponds to increased GSH and GSSG and indicates a plausible mechanism for resistance to EtOH-induced oxidative stress. In a previous report, expression of GSTm6 was decreased in PTEN^LKO^ mice whereas we find no significant change [14]. In that report, array analysis was performed at 10 weeks of age and the authors hypothesize that downregulation of GSTm6 may contribute to an increase in inflammation that occurs after 10 weeks of age. Our data originated from 18 week old PTEN^LKO^ mice and demonstrate no significant change in GSTm6 expression in PTEN^LKO^ PF groups when compared to PTEN^f/f^ PF groups. In the previous study, analysis did not report differences in other GST isoforms. In this study, we find that GSTa1/2/3/4/5 and GSTm3 are all upregulated in PF PTEN^LKO^ mice when compared to PF PTEN^f/f^ mice indicating an enhanced response to diet induced inflammation. Chronic EtOH challenge however, decreases GSTm6 expression and decreased GSTA isoforms suggesting that inflammation may be decreased. In a recent publication, PTEN^LKO^ mice were protected against endotoxemia [21]. In that study the authors determined that heme oxygenase was upregulated by PPARγ. We do not see HO-1 upregulation in this study but we do see increased PPARγ in the PTEN^LKO^ model [15]. A difference between our study, is that Guenzl et al, used mice younger than 12-weeks for their studies and there are some reports that suggest that 12 weeks of age is necessary for the full Cre-recombination and PTEN deletion to take effect [12–14, 45].

Interestingly, using the same model, 6-weeks consumption of alcohol and WT SV and C57BL/6J mice, protein adducts are increased in the periportal region [19, 34, 46]. This also occurs in the PTEN^f/f^ controls. In this study, protein-SSG is only increased around the central venous region in PTEN^f/f^ mice but is increased panlobularly with EtOH in the PTEN^LKO^ model. This suggests that glutathionylation may also prevent carbonylation of proteins. Recent evidence suggests that GSTμ by its protein-SSG regulatory function, exerts a positive influence against ER stress [47]. In support of this mechanism, both GSTμ and protein-SSG are elevated in PF PTEN^LKO^ mice when compared to PF PTEN^f/f^ controls. Increased GST activity exerts a protective effect in NASH [48]. Post-translational modification of cysteine residues occurs in both glutathionylation and carbonylation [48–51]. Increased glutathionylation would protect critical cysteine residues by preventing carbonylation in a reversible mechanism. Future studies will be necessary to fully elucidate the impact of increased glutathionylation and inflammation in PTEN^LKO^ mice chronically fed EtOH and to determine clinical relevance.

In conclusion, this study examined the effects of chronic EtOH consumption in the background of constitutively activated *de novo* lipogenesis, increased steatosis and increased cellular respiration. The data obtained provide new insight into the hepatocellular outcomes of preexisting steatosis due to increased Akt activation during alcohol consumption [19]. In PTEN^LKO^ mice, EtOH consumption did not exacerbate hepatocellular damage suggesting that an environment of increased hepatocellular glutathione concentration may be protective. We hypothesize that reduced injury evident in this study is in part from enhancement of protective oxidative stress responses due to increased cellular respiration occurring during constitutive Akt activation (PTEN^LKO^) (**S1 Fig**). This preexisting condition in PTEN^LKO^ mice results in an alteration of glutathione homeostasis creating a compensatory hepatocellular environment primed to mitigate increased oxidative stress and hepatocellular damage by EtOH. Alternatively, in the background of increased *de novo* lipogenesis, the addition of EtOH mitigates the effects of a high fat diet. Additional studies will be necessary to determine the sustainability these compensatory mechanisms under conditions of long-term ethanol ingestion and to determine proteins downstream of PTEN that contribute to the protective effect.

## Supporting Information

S1 Fig Effects of PTEN^LKO^ and EtOH on hepatic PTEN and Akt expression. (A) Cytosolic extracts from PF and EtOH-fed PTEN^f/f^/PTEN^LKO^ groups were analyzed via SDS PAGE, Western blotted and probed for PTEN, pSer^473^ Akt, and total Akt. (B) Quantification of the Western blots presented in S1A Fig (actin normalized). Data are means± SEM as analyzed by students t-test (PF/EtOH) and two-way ANOVA with a Bonferroni *post hoc* analysis (PTEN^f/f^ group compared to PTEN^LKO^ group) (N = 3 mice/group (*p<0.05, ***p<0.001)).
(DOCX)

S1 Table Pathway analysis of up/downregulated oxidative stress proteins in PF/EtOH fed PTEN^f/f^/PTEN^LKO^ mice. Proteins identified in Fig 4 were examined using KEGG pathway analysis as previously described [49].
(XLSX)

## Abbreviations

4-HNE: 4-Hydroxynonenal
ALD: Alcoholic Liver Disease
ALT: Alanine aminotransferase
ANOVA: Analysis of variance
BEC: Blood ethanol concentration
Cyp2E1: Cytochrome P450 2E1
DNL: *De novo* lipogenesis
GCLC: Glutathione cysteine ligase
GSH: Reduced glutathione
GSS: Glutathione synthetase
GSSG: Glutathione disulfide
GSTA4: Glutathione S-transferase A4
MDA: Malondialdehyde
NAFLD: Nonalcoholic fatty liver disease
PF: Pair-fed
PTEN: Phosphatase and tensin homolog on chromosome 10
PTEN^f/f^: PTEN mice with floxed alleles but without Cre-Recombinase
PTEN^LKO^: Liver-specific PTEN deletion mice
PtdIns: Phosphatidylinositol
SEM: Standard error of the mean
