# Supplementary material for: Liver-Specific Deletion of Phosphatase and Tensin Homolog Deleted on Chromosome 10 Significantly Ameliorates Chronic EtOH-Induced Increases in Hepatocellular Damage
Source: PLoS One. 2016 Apr 28;11(4):e0154152. doi: 10.1371/journal.pone.0154152 (PMC4849752; doi:10.1371/journal.pone.0154152)
Supplement: S1 Fig — (A) Cytosolic extracts from PF and EtOH-fed PTENf/f/PTENLKO groups were analyzed via SDS PAGE, Western blotted and probed for PTEN, pSer473 Akt, and total Akt. (B) Quantification of the Western blots presented in S1A Fig (actin normalized). Data are means± SEM as analyzed by students t-test (PF/EtOH) and two-way ANOVA with a Bonferroni post hoc analysis (PTENf/f group compared to PTENLKO group) (N = 3 mice/group (*p<0.05, ***p<0.001)). (DOCX) [file pone.0154152.s001.docx]

**Supporting Information**

**This is the Fig S1 legend: Fig. S1. Effects of PTEN^LKO^ and EtOH on hepatic PTEN and Akt expression.** (A) Cytosolic extracts from PF and EtOH-fed PTEN^f/f^/PTEN^LKO^ groups were analyzed via SDS PAGE, Western blotted and probed for PTEN, pSer^473^ Akt, and total Akt. (B) Quantification of the Western blots presented in **S1A Fig** (actin normalized). Data are means± SEM as analyzed by students t-test (PF/EtOH) and two-way ANOVA with a Bonferroni *post hoc* analysis (PTEN^f/f^ group compared to PTEN^LKO^ group) (N=3 mice/group (*p<0.05, ***p<0.001)).
